# Malnutrition in gastrointestinal cancer manifests before systemic therapy and is associated with fatigue and reduced physical quality of life

**DOI:** 10.1093/oncolo/oyag028

**Published:** 2026-02-03

**Authors:** Mats L Wiese, Lena Schwarz, Sabrina von Rheinbaben, Sebastian Schmidt, Markus Blaurock, Jan Krönke, Markus M Lerch, Luzia Valentini, Ali A Aghdassi

**Affiliations:** Department of Food Nutrition Facilities, University of Applied Sciences Münster, Münster, Germany; Department of Medicine A, University Medicine Greifswald, Greifswald, Germany; University Children’s Hospital Zurich, University of Zurich, Zurich, Switzerland; Institute of Evidence-based Dietetics (NIED), University of Applied Sciences Neubrandenburg, Neubrandenburg, Germany; Department of Medicine C, University Medicine Greifswald, Greifswald, Germany; Department of Medicine A, University Medicine Greifswald, Greifswald, Germany; Department of Otorhinolaryngology, Head and Neck Surgery, University Medicine Greifswald, Greifswald, Germany; Department of Medicine C, University Medicine Greifswald, Greifswald, Germany; University Hospital, Ludwig Maximilians University Munich, Munich, Germany; Institute of Evidence-based Dietetics (NIED), University of Applied Sciences Neubrandenburg, Neubrandenburg, Germany; Department of Medicine A, University Medicine Greifswald, Greifswald, Germany

**Keywords:** malnutrition, cachexia, gastrointestinal cancer, quality of life, fatigue, patient-reported outcomes

## Abstract

**Background:**

There is a high malnutrition risk in patients with gastrointestinal tumors. Yet it is unknown when malnutrition manifests and how changes in nutritional status are related to quality of life and fatigue at different stages of oncologic therapy.

**Patients and Methods:**

In a prospective observational study, we recruited patients with initial diagnosis of any gastrointestinal tumor requiring systemic therapy and respective patients already receiving treatment. Subjects underwent comprehensive nutritional assessment at enrollment and after 3 months. In addition, patients reported data on physical activity (IPAQ-SF), quality of life (SF-12), and fatigue (EORTC QLQ-FA12). Besides baseline associations, relations between changes in nutritional status and patient-reported outcomes during treatment were analyzed.

**Results:**

We included 66 patients (mean(±SD) age: 62.1(±10.6) yrs.; 68% male), of which 29 had received initial diagnosis and 37 were already undergoing treatment. Baseline clinical characteristics and nutritional status were comparable between groups. With 88% of patients, GLIM-defined malnutrition was highly prevalent at baseline and associated with fatigue, reduced physical activity, and quality of life (*P* < .01, respectively). Among 36 study completers, only minor fat mass (*P* = .033) and progressed weight loss (*P* = .025) indicated further nutritional deterioration. Development of cachexia, but not malnutrition or sarcopenia, during treatment was associated with impaired patient-reported outcomes, ie, higher fatigue (rho = 0.400; *P* = .019) and lowered physical activity (rho=-0.423; *P* = .013).

**Conclusion:**

Most patients with gastrointestinal cancer are malnourished already at diagnosis. Impaired nutritional status is closely linked with reduced quality of life and fatigue, especially their physical components. Trials are warranted to test whether optimized nutrition support can halt further aggravation during treatment.

Implications for Practice Patients with gastrointestinal cancer have a high risk of malnutrition, but it is unclear at what time point of disease malnutrition develops and how this condition is related to quality of life and fatigue. This work shows that most patients are already malnourished at the time of diagnosis and that systemic cancer treatment only leads to a little further nutritional decline. As impaired nutritional status was closely related to reduced physical quality of life and higher fatigue, this study suggests that optimized nutritional treatment starting with the initial diagnosis could lead to improvement of these symptoms in persons with gastrointestinal cancer.

## Introduction

Patients with gastrointestinal cancer have a high malnutrition risk.^1–3^ Malnutrition results from reduced food intake due to loss of appetite or pain, malassimilation of nutrients, and tumor-driven systemic inflammation. Besides disease itself, also tumor therapy can foster deteriorations in nutritional status.^4^ Reported prevalences of malnutrition in patients with gastrointestinal cancer are between 13% and 98%.^5^ The number of malnourished patients differs not only by tumor location but also by the diagnostic criteria applied.^6^^,^^7^ Use of inconsistent definitions hampers both research and especially treatment of malnutrition. Moreover, the presence of overlapping, often coexisting conditions, ie, malnutrition, sarcopenia, and cachexia, presents an additional challenge in that regard.^8^^,^^9^

Besides the unknown exact prevalence, it remains unclear at what stage of disease malnutrition develops and how patients’ nutritional condition changes during therapy. Systemic tumor therapy specifically is known to have side-effects, eg, nausea and vomiting, that may exacerbate dietary deficits.^10^^,^^11^ Conversely, a good treatment response could improve disease-related gastrointestinal symptoms and thus attenuate wasting of body reserves.

The consequences of alterations in nutritional status during systemic tumor therapy are not fully understood. Some studies have shown worse treatment tolerance and survival in malnourished patients with gastrointestinal cancer undergoing systemic therapy.^12–14^ However, the relationship between changes in nutritional status and patient-reported outcomes before and during such treatment is unknown. Quality of life and fatigue, in that regard, are particularly relevant outcomes in this patient group, as they give insights into the patient’s experience and can help to provide personalized care along the patient’s journey. In case these patient-reported outcomes are associated with changes in nutritional status, quality of life and fatigue could present supportive targets in multimodal therapy.

Closing the aforementioned research gaps regarding the prevalence and onset of malnutrition as well as its relationship with these patient-reported outcomes could aid in optimizing therapy and symptom burden. Therefore, in the present work, we comprehensively investigated the trajectory of nutritional status and its interrelation with quality of life and fatigue in patients with gastrointestinal cancer during systemic therapy.

## Patients and methods

### Study design and population

We performed a monocentric, observational study in which we prospectively enrolled patients with gastrointestinal cancer requiring systemic drug-based therapy. Patients were recruited at University Medicine Greifswald, a tertiary referral cancer center located in northeast Germany, between June 2020 and December 2022.

All patients aged 18 years or older who (1) had received an initial diagnosis of any malignant tumor of the gastrointestinal tract and planned to receive or (2) with ongoing systemic therapy for such malignancy were approached for study participation. We excluded subjects who were pregnant, had a history of any other malignant tumor disease, or were unable to provide informed consent.

Assessments of nutritional status, quality of life, and fatigue were performed at baseline and after 3 months. The study was approved by the Institutional Review Board at University Medicine Greifswald (internal registration number: BB 071/20) and registered at clinicaltrials.gov (NCT04476082). All study-related procedures were carried out in accordance with the Declaration of Helsinki.

### Clinical and patient data

Personal and disease-related data were obtained via standardized interview or extracted from the patient files. Furthermore, we employed the International Physical Activity Questionnaire short form (IPAQ-SF) to inquire about patients’ physical activity during the last 7 days preceding hospital attendance. Routine and nutritional blood parameters were retrieved from the clinical information system at every time point.

### Assessment of nutritional status

The international consensus criteria by the Global Leadership Initiative on Malnutrition (GLIM) were applied to diagnose malnutrition.^15^ We performed body composition analysis with the seca mBCA 525 (seca, Hamburg, Germany), an 8-electrode, phase-sensitive, segmental bioelectrical impedance analysis (BIA) device. The measurement was conducted following a standardized protocol with patients in a supine position using adhesive gel electrodes placed on the dorsal surfaces of hand, wrist, ankle, and foot. All measurements were taken before beginning intravenous infusions. Sex-specific thresholds to confirm reduced muscle mass criterion were applied as previously described^16^: Moderate reduction was defined as a skeletal muscle mass index (SMMI) of the general population mean minus one standard deviation (♀: 6.68 kg/m^2^, ♂: 8.97 kg/m^2^), and severe reduction as the general population mean minus two standard deviations (♀: 5.86 kg/m^2^, ♂: 8.14 kg/m^2^). The etiologic criterion of disease burden/inflammation was considered fulfilled by all patients due to their malignant condition, usually associated with mild to moderate chronic inﬂammation.^17^ Diagnosis of sarcopenia was performed according to the European Working Group on Sarcopenia in Older People 2 (EWGSOP2) criteria.^18^ To detect impaired muscle strength, we measured handgrip strength using the Jamar Plus+ Digital Hand Dynamometer (Patterson Medical, Warrenville, IL, USA). Three measurements were taken with the patients seated, elbow in 90° flexion, and the wrist in a neutral position using their dominant hand. The maximum strength value was used for analysis. For confirmation of sarcopenia in patients with reduced handgrip strength (♀: <16 kg, ♂: <27 kg) the same SMMI cut-offs as for diagnosis of severe malnutrition were applied. Cachexia was diagnosed based on the Fearon criteria, considering weight loss, reduced BMI, and muscle mass.^19^

### Quality of life and fatigue

To assess patients’ quality of life, we employed the German version of the 12-Item Short-Form Health Survey (SF-12). The SF-12 is a validated questionnaire covering a mental and a physical component of health-related quality of life.^20^^,^^21^ To measure fatigue, the EORTC QLQ-FA12 questionnaire^22^ was used. Patients answered both questionnaires upon enrollment and after 3 months.

### Statistical analyses

Descriptive continuous data are presented as mean (±SD) or median (IQR) for normally and non-normally distributed variables, respectively. Categorical data are given as absolute numbers (n) and relative frequencies (%). To test differences between two groups, we used Chi-squared test for categorical data and the two-sided t-test or the Mann-Whitney U test for normally and non-normally distributed continuous variables. Accordingly, the Fisher’s exact test, one-way ANOVA, or the Kruskal-Wallis test was employed when comparing more than two groups. In case the ANOVA or the Kruskal-Wallis test yielded a significant result, we ran post-hoc tests with Bonferroni correction to determine differences between individual groups.

For longitudinal analyses, the McNemar test was used to investigate changes in patients’ status of malnutrition, sarcopenia, and cachexia. We applied the paired samples Wilcoxon or t-test to determine significant changes in continuous variables depending on the normality of data distribution. Spearman correlation coefficients were calculated for testing the relation of alterations in parameters of nutritional status, quality of life, fatigue, and physical activity during the 3 months of therapy.

Analyses and graphical visualization were performed using IBM SPSS Statistics for Windows version 28 (IBM Corp., Armonk, NY, United States) or R software (R Core Team, Vienna, Austria) for statistical computing (version 4.2.0). A two-sided *P*-value of less than .05 defined statistical significance.

## Results

### Patient selection and characteristics

A total of 66 patients were initially enrolled in this study. Basic patient and clinical characteristics are presented in Table 1. Twenty-nine subjects were enrolled at initial diagnosis of a malignant gastrointestinal tumor before receiving systemic therapy; 37 were already undergoing such treatment. Median time from diagnosis was 4 months in the latter group. There were no significant differences in age (64.2 (±8.0) yrs vs. 60.5 (±12.1) yrs; *P* = .160) and sex (76% male vs. 62% male; *P* = .206) between groups. Clinical characteristics were also comparable between both patient populations. Pancreatic and colorectal cancer were the most common entities. The tumor was metastasized in around half the cases, and treatment intention was mainly palliative. Most patients had an ECOG performance status of 0 or 1. Blood parameters were mainly comparable but indicated slightly higher inflammatory status in patients at initial diagnosis (Table S1).

**Table 1.** Baseline demographic and clinical patient characteristics.

	Total (n = 66)	Initial diagnosis (n = 29)	Undergoing systemic therapy (n = 37)	*P*-value
**Age, years**	62.1 (±10.6)	64.2 (±8.0)	60.5 (±12.1)	.160
**Male, n (%)**	45 (68)	22 (76)	23 (62)	.206
**Tumor entity, n (%)**				.269
** Pancreas**	21 (32)	9 (31)	12 (32)	
** Colorectal**	23 (35)	8 (28)	15 (41)	
** Gastric**	10 (15)	6 (21)	4 (11)	
** Esophagus**	4 (6)	3 (10)	1 (3)	
** Liver**	2 (3)	2 (7)	-	
** Bile duct**	5 (8)	1 (3)	4 (11)	
** Neuroendocrine**	1 (2)	-	1 (3)	
**Metastasized, n (%)**	38 (58)	17 (59)	21 (57)	.879
**Treatment intention, n (%)**				.206
** Curative**	24 (36)	13 (45)	11 (30)	
** Palliative**	42 (64)	16 (55)	26 (70)	
**ECOG performance status, n (%)**				.615
** 0**	28 (42)	14 (48)	14 (38)	
** I**	24 (36)	8 (28)	16 (43)	
** II**	10 (15)	5 (17)	5 (14)	
** III**	4 (6)	2 (7)	2 (5)	
**Time since initial diagnosis, months**	1.9 (4.3)	0 (0)	4 (6)	**<.001**

Data are presented as mean (±SD), median (IQR), or n (%).

Differences between groups for continuous data were tested by two-sided t-test or Mann–Whitney U test depending on the normality of data distribution.

Differences between groups for categorical data were tested by Chi-squared or Fisher’s exact test.

### Baseline nutritional status

Nutritional status was overall similar between both groups at enrollment (Table 2). Total malnutrition prevalence was 88%, with 83% and 92% being malnourished among patients at diagnosis or undergoing treatment, respectively. There were no significant differences in phenotypic or etiologic malnutrition criteria. In both groups, severe malnutrition was observed in most patients (45% vs. 49%). Body composition was comparable between groups (*P* > .05), whereas overall weight loss was higher in patients already undergoing therapy (14.3% vs. 9.0%; *P* = .028) (Figure S1). Patients at initial diagnosis were more likely to have concomitant cachexia than those under treatment (79% vs. 49%; *P* = .011). By contrast, sarcopenia was extremely rare in both groups. The overlap between diagnoses of malnutrition, cachexia, and sarcopenia is illustrated in Figure S2.

**Table 2.** Baseline nutritional status of patients.

	Total (n = 66)	Initial diagnosis (n = 29)	Undergoing systemic therapy (n = 37)	*P*-value
**Nutritional Risk**				
** NRS-2002, pts**	3 (2)	3 (2)	3 (2)	.314
** NRS-2002 ≥ 3 pts, n (%)**	38 (58)	19 (66)	19 (51)	.248
**Reduced food intake, n (%)**	42 (64)	18 (62)	24 (65)	.815
**Malassimilation/Chronic gastrointestinal condition, n (%)**	21 (32)	8 (28)	13 (35)	.512
**Low body mass index, n (%)**	11 (17)	6 (21)	5 (14)	.515
**Weight loss, n (%)**	50 (76)	23 (79)	27 (73)	.551
**Reduced muscle mass, n (%)**	32 (48)	14 (48)	18 (49)	.976
**Malnutrition, n (%)**				.612
** No**	8 (12)	5 (17)	3 (8)	
** Moderate**	27 (41)	11 (38)	16 (43)	
** Severe**	31 (47)	13 (45)	18 (49)	
**Cachexia, n (%)**	41 (62)	23 (79)	18 (49)	**.011**
**Sarcopenia, n (%)** ^a^	1 (2)	1 (3)	0 (0)	.453

Data are presented as n (%) or median (IQR).

Differences between groups for continuous data were tested by Mann–Whitney U test.

Differences between groups for categorical data were tested by Chi-squared or Fisher’s exact test.

a n = 35 for patients undergoing systemic therapy due to missing values.

### Relation of nutritional status with quality of life, fatigue, and physical activity at baseline

**Figure 1.**
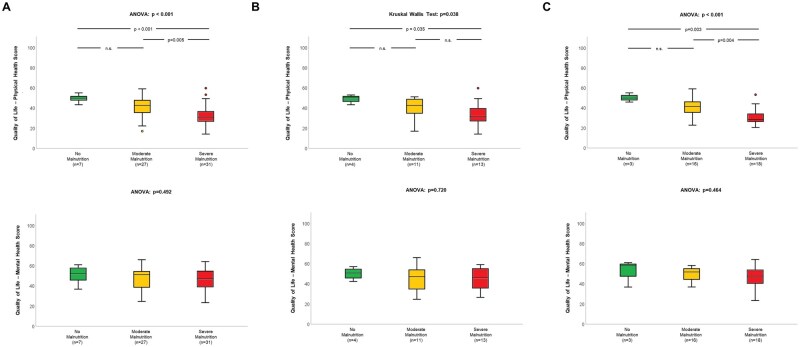
Relation of nutritional status and quality of life in all patients with gastrointestinal cancer (A) and subgroups of persons at initial diagnosis (B) or already undergoing systemic treatment (C).

**Figure 2.**
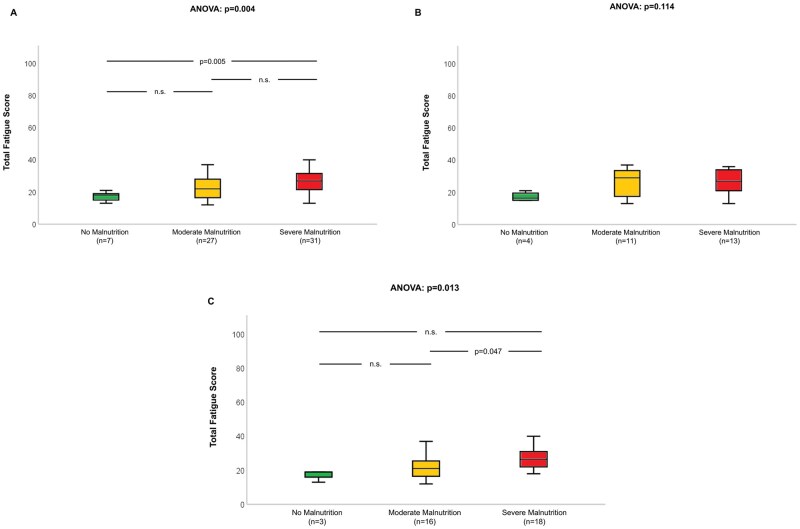
Relation of nutritional status and fatigue in all patients with gastrointestinal cancer (A) and subgroups of persons at initial diagnosis (B) or already receiving systemic treatment (C).

When we investigated the relation between baseline nutritional status and quality of life, we found malnutrition to be associated with impaired physical—but not mental—health score (Figure 1A-C). The link of malnutrition with decreased physical quality of life was seen in the total population (*P* < .001; Figure 1A), as well as the individual groups of patients at initial diagnosis (*P* = .038; Figure 1B) or those undergoing treatment (*P* < .001; Figure 1C), and more pronounced with increasing severity of malnutrition. Compromised nutritional status was associated with fatigue in the total population (*P* = .004; Figure 2A) and in subjects already undergoing therapy (*P* = .013; Figure 2C) but not those at initial diagnosis (*P* = .114; Figure 2B). For fatigue subscales, we found the association with malnutrition primarily related to physical fatigue (Table S2). Regarding physical activity, we further observed malnutrition to be associated with reduced overall activity level (*P* = .005), lower metabolic equivalent of task hours (*P* = .001) as well as more time spent sitting (*P* = .016) in the total population. Considering the two subgroups, significant relations were only seen among patients already receiving treatment but not those at initial diagnosis (Table 3).

**Table 3.** Relation between nutritional status and physical activity.

	Total (n = 65)^a^			
	No malnutrition (n = 7)	Moderate malnutrition (n = 27)	Severe malnutrition (n = 31)	^b^ *P*-value
**Activity level, n (%)**				**.005**
** Low**	0 (0)	4 (15)	15 (48)	
** Moderate**	2 (29)	7 (26)	9 (29)	
** High**	5 (71)	16 (59)	7 (23)	
**MET hours/week**	71.1 (73.8)	56.0 (86.7)	11.5 (47.6)	**.001**
**Sitting hours/day**	6.0 (3.0)	5.0 (2.0)	8.0 (7.0)	**.016**

	Initial diagnosis (^a^n = 28)			
	No malnutrition (n = 4)	Moderate malnutrition (n = 11)	Severe malnutrition (n = 13)	^b^ *P*-value
**Activity level, n (%)**				.382
** Low**	0 (0)	2 (18)	4 (31)	
** Moderate**	1 (25)	3 (27)	6 (46)	
** High**	3 (75)	6 (55)	2 (23)	
**MET hours/week**	107.0 (179.8)	51.1 (78.0)	23.1 (60.7)	.119
**Sitting hours/day**	4.0 (2.8)	5.0 (3.5)	7.5 (5.8)	.062

	Undergoing chemotherapy (n = 37)			
	No malnutrition (n = 3)	Moderate malnutrition (n = 16)	Severe malnutrition (n = 18)	*P*-value^b^
**Activity level, n (%)**				**.015**
** Low**	0 (0)	2 (13)	11 (61)	
** Moderate**	1 (33)	4 (25)	3 (17)	
** High**	2 (67)	10 (63)	4 (22)	
**MET hours/week**	55.2 (–)	75.8 (69.7)	8.9 (49.4)	**.004**
**Sitting hours/day**	6.0 (–)	5.3 (2.0)	8 (7.3)	.087

MET, metabolic equivalent of task.

Data are presented as median (IQR) unless indicated otherwise.

a One patient did not complete the food frequency questionnaire and was excluded from analysis.

b Differences between groups of nutritional status were tested using Fisher’s exact test for categorical variables and Kruskal-Wallis test for continuous variables.

### Changes in nutritional status and blood parameters

**Figure 3.**
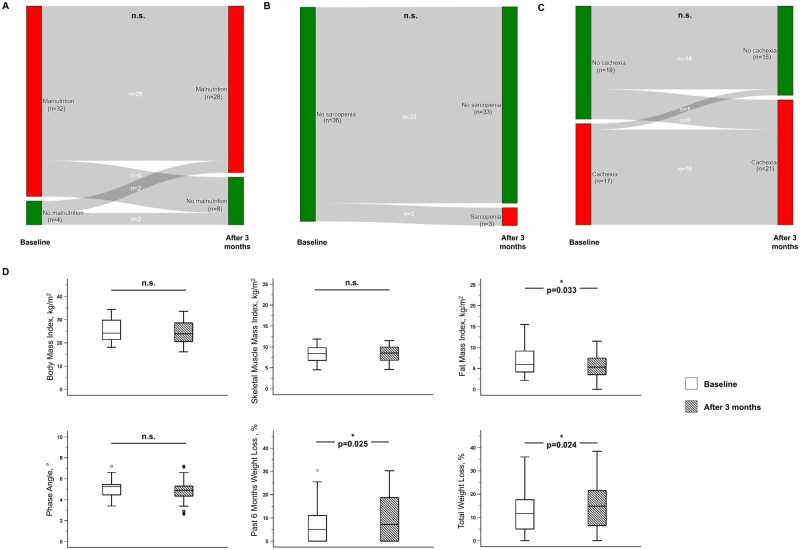
Changes in nutritional status of 36 patients with gastrointestinal cancer during 3 months of therapy indicated by prevalence of malnutrition (A), sarcopenia (B), or cachexia (C) as well as body composition and weight loss parameters (D). Differences between time points for categorical data were tested by the McNemar test. Wilcoxon or t-test, depending on the normality of data distribution. Differences between time points for continuous data were tested by paired samples Wilcoxon or t-test depending on the normality of data distribution.

Of the 66 patients initially enrolled, 36 participated in the follow-up investigation after 3 months. Among these subjects, we found no major changes in nutritional status. Prevalences of malnutrition, sarcopenia, and cachexia were comparable at both time points (Figure 3A-C). Regarding body composition, we observed a significant decrease in fat mass (*P* = .033) but no changes in BMI, SMMI, and phase angle (*P* > .05). Meanwhile, weight loss in the past 6 months as well as total weight loss significantly increased (*P* = .025 and *P* = .024, respectively) (Figure 3D). All routine and nutritional blood parameters were unchanged to baseline levels (Table S3).

Comparing baseline characteristics between the subjects who completed the study and those who dropped out, we found overall similar demographic and patient characteristics as well as nutritional status (Tables S4-6). Completers were more often already undergoing therapy, receiving curative treatment, and had longer prior treatment duration, though; while dropouts showed higher prevalence of reduced food intake as well as cachexia and had greater weight loss in the past 6 months.

### Relation of changes in nutritional status and quality of life, fatigue, and physical activity during 3 months of therapy

**Figure 4.**
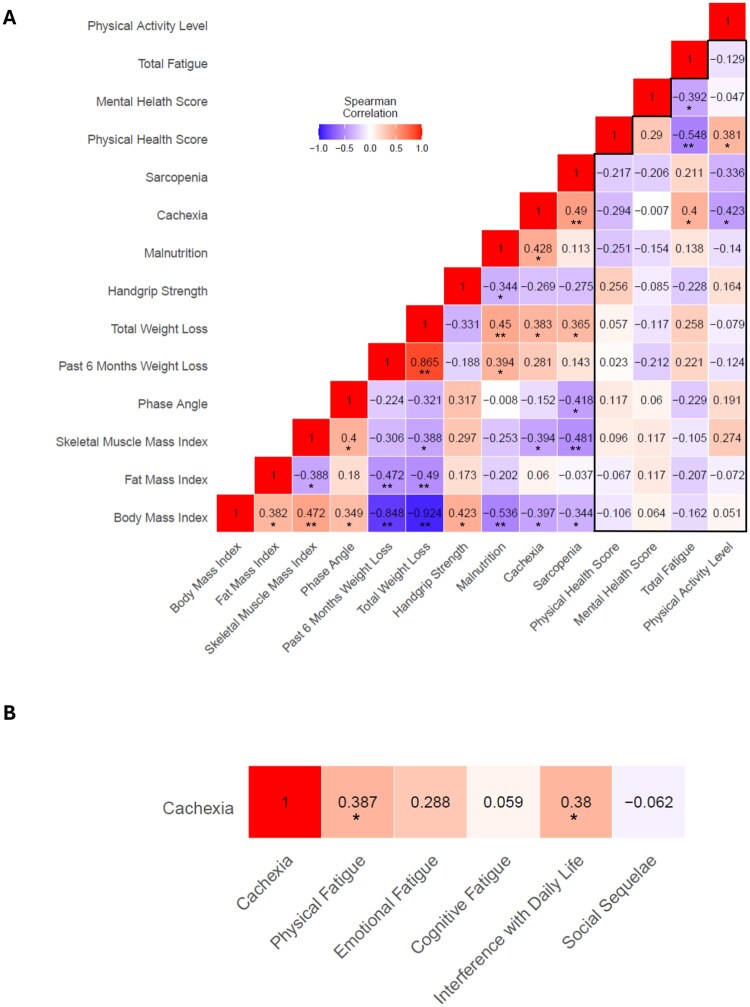
Spearman correlation coefficients illustrating the relations of changes in parameters of nutritional status, quality of life, fatigue, and physical activity (A) as well as links between development of cachexia and fatigue subscales (B) in 36 patients with gastrointestinal cancer during 3 months of therapy. ^*^P < .05; ^**^P < .01.

When we analyzed the correlations of changes in indicators of nutritional status, quality of life, fatigue, and physical activity, we observed distinct relationships (Figure 4A). While there were several significant correlations between individual diagnostic parameters, alterations in none of these nutritional indicators were linked to changes in quality of life, fatigue, or physical activity. Likewise, newly diagnosed malnutrition or sarcopenia was related to changes in selected diagnostics parameters but did not correlate with a decline in quality of life, fatigue, or physical activity (*P* > .05, respectively). Conversely, new-onset cachexia during the 3 months of therapy was not only linked to greater total weight loss as well as declines in BMI and SMMI but also lower physical activity (rho=-0.423; *P* = .013) and increased fatigue (rho = 0.400; *P* = .019). Additional analyses testing the relation between cachexia and fatigue subscales revealed significant correlations with physical fatigue (rho = 0.387; *P* = .024) and interference with daily life (rho = 0.380; *P* = .027) (Figure 4B).

## Discussion

In this work, we show that in patients with gastrointestinal cancer, malnutrition is commonly already manifested at diagnosis, whereas subsequent systemic therapy leads to minor further decline in nutritional status. Moreover, we found impaired nutritional status directly linked with reduced quality of life and fatigue, especially in terms of physical impairment.

Our study adds to existing knowledge, as it provides novel insights into the dynamics of nutritional status during systemic therapy and its interrelation with changes in relevant patient-reported outcomes.

Regarding the onset of malnutrition in patients with gastrointestinal tumors, we found comparable nutritional status among subjects at initial diagnosis and those already undergoing systemic therapy. Notably, except for overall weight loss, phenotypic malnutrition criteria were comparable, and newly diagnosed subjects showed an even higher presence of cachexia. Together with its high prevalence, our results imply that malnutrition mainly develops before cancer diagnosis and not as a result of anti-tumor treatment. Our longitudinal analyses showing little further deterioration after 3 months support this argumentation as well as our finding of higher CRP levels in patients with an initial diagnosis seen at baseline, given inflammation is a known driver of compromised nutritional status in patients with cancer.^4^^,^^9^ A limited number of previous studies have shown inconclusive results regarding changes in nutritional status during systemic therapy.^23–26^ While 2 studies observed a nutritional decline during chemotherapy,^23^^,^^24^ 2 other trials found unchanged nutritional status despite reduced food intake or the development of nutrition-related symptoms.^25^^,^^26^ Importantly, these studies were heterogeneous in design, eg, in terms of investigated tumor entities, follow-up duration, and nutritional status parameters. The work by Rasschaert et al.^24^ was the only one to apply the GLIM criteria. However, they only describe a numerical increase in malnutrition prevalence and in a population not limited to gastrointestinal cancers. By contrast, another study, including only patients with colorectal cancer^25^ found no changes in nutritional status. However, the study duration was rather short in that investigation, being limited to a single chemotherapy cycle. Considering inconclusive previous research, our work is of particular relevance to the treatment of patients with gastrointestinal cancer, suggesting most cases require continuous nutritional support from the time of diagnosis.

While malnutrition prevalence in patients was high in both groups, we observed a limited overlap with cachexia and sarcopenia. Especially, the latter was rarely seen in patients both at the baseline and follow-up investigations. Although concomitant cachexia was found more commonly in malnourished patients, our results suggest different dynamics of both conditions during systemic therapy. While malnutrition was successfully overcome in 6 out of 32 patients, only 1 in 17 individuals became non-cachectic during follow-up. Further, prevalence of cachexia increased, whereas the number of patients with malnutrition declined.

However, in that regard, it should be noted that the GLIM criteria provide a framework for evaluating cachexia, defined as malnutrition related to chronic disease with inflammation. Hence, a strong overlap could be assumed. Nevertheless, a recent meta-analysis demonstrated that operationalization of the GLIM criteria varies between studies and that the optimal combination of phenotypic and etiologic criteria to determine clinical outcomes has yet to be defined.^27^ Operationalization of the criteria will also impact observed prevalences, which is why the diagnostic approach chosen in our work deserves discussion. Especially, the phenotypic criterion of reduced muscle mass and the etiologic inflammation criterion have been debated, which prompted the GLIM to provide further guidance for assessment.^17^^,^^28^ Although guidance papers were published after our study started, the appropriateness of our diagnostic approaches is supported by these publications.

Regarding muscle mass assessment, use of BIA, despite having limitations, is endorsed by the GLIM when appropriate expertise in its use is given.^28^ The validity and prognostic value of BIA-derived body composition parameters in patients with cancer have been demonstrated repeatedly.^29–31^ While computed tomography scans, frequently used for muscle mass assessment, might be available in most cancer patients, there are limitations to this method as well, eg, heterogeneity in protocols and technical settings.^28^ Likewise, our assumption that all patients fulfilled the inflammation criterion is supported by the GLIM guidance on assessment of this criterion.^17^ A recent prospective investigation in patients with advanced cancer identified inflammation to be the most important etiologic criterion for predicting survival.^32^ Therefore, it is important to acknowledge heterogeneity in terms of assessment of this criterion. Our results based on CRP levels support that inflammation was present in most patients with malnutrition. Yet, other inflammatory parameters, ie, modified Glasgow Prognostic Score or Neutrophil-to-Lymphocyte Ratio, have been suggested to hold greater prognostic value.^32^ The identification of the ideal combination of phenotypic and etiologic criteria in patients with cancers warrants further research. Meanwhile, the observed prevalences of malnutrition and cachexia in our study should be interpreted in view of the applied diagnostic approaches.

In contrast to our results, another recent study^33^ reported significantly better agreement between malnutrition, sarcopenia, and cachexia, with prevalences being 60%, 53%, and 57%, respectively. However, this work, again, also included patients with other cancers besides gastrointestinal origin, and all subjects were 70 years or older. In addition, that study employed multiple diagnostic tools, including screening instruments, for diagnosing these conditions. These methodological differences likely explain the discrepant results, especially regarding sarcopenia, and emphasize the additional value of our work. There is consensus that sarcopenia and cachexia present overlapping syndromes, which, despite intersecting with malnutrition and resembling its phenotype, yet have distinct etiologic factors.^34^^,^^35^ Our findings regarding the apparently different patterns of onset and dynamics in patients with gastrointestinal cancer highlight the importance of dissecting these conditions using standardized and validated diagnostic approaches.

In that regard, the observed associations of these syndromes with patient-reported outcomes, namely quality of life, fatigue, and physical activity, are noteworthy. Prevalence of malnutrition, the primary outcome measure of our study, was associated with impaired physical quality of life, fatigue, and reduced physical activity at baseline. These findings concur with earlier research addressing these relations in cross-sectional designs. A link between impaired nutritional status and reduced quality of life as well as fatigue has repeatedly been shown in patients with gastrointestinal cancer, despite the use of various different tools for assessment of nutritional status and quality of life or fatigue.^36–38^ Our results still provide further insights in this context. Using validated and standardized assessment instruments for all these parameters, we show impaired nutritional status to be primarily related to the physical components of these constructs. Reduced physical activity, which we found mutually to be associated with malnutrition, likely reflects these relations. Interestingly, a link between fatigue and malnutrition was only seen in subjects already receiving therapy. This finding suggests that systemic treatment could have an important mediating effect in this relation, which is supported by previous research as well. For instance, a recent study among 135 advanced cancer patients, mostly with gastrointestinal tumors, admitted to a specialized home palliative care program showed an independent association between fatigue and malnutrition.^39^ Evidence from longitudinally designed trials, which could help elucidate the relation of nutritional status, anticancer treatment, quality of life, and fatigue is limited, though. A recent study by Hanna et al.^40^ reported that in patients with newly diagnosed upper gastrointestinal cancer, malnutrition risk independently predicted quality of life at baseline as well as after 3 and 6 months. Interestingly, this study found no association with emotional function, either. Few other studies investigated changes in quality of life and fatigue in patients with gastrointestinal cancer following provision of nutritional support.^41–44^ All but one study showed improved quality of life or fatigue resulting from the intervention. This trial^42^ tested the effect of early, intensified remote nutritional counselling during anticancer treatment in patients with upper gastrointestinal cancer. Besides unchanged quality of life and fatigue, the intervention also did not lead to improved nutritional status, suggesting that such remote interventions might not be a suitable approach in these patients overall. However, it should be considered that improved quality of life and fatigue may not necessarily be accompanied by concurrent changes in all nutritional indicators. For instance, one retrospective analysis^43^ investigated changes in quality of life and its symptoms in patients attending a clinical service for cancer cachexia. While benefits in quality of life and fatigue symptoms were observed, body weight and muscle function remained unchanged. Admittedly, the multimodal treatment provided in this study also comprised other than nutritional interventions. However, these findings do not necessarily imply that provision of nutritional support has no benefit regarding quality of life or fatigue in patients with gastrointestinal cancer. In our own investigation, we found cachexia rather than malnutrition or other nutritional parameters to be associated with fatigue and reduced physical activity. Both cachexia and fatigue are complex syndromes that despite being interrelated with nutritional status, require multidisciplinary intervention, including exercise and psychosocial care.^9^^,^^45^ Arguably, providing only nutrition support to patients with gastrointestinal cancer might not suffice to halt a decline in quality of life and progression of fatigue. Nevertheless, more research is needed to clarify the relevance of the different modalities in that regard.

## Limitations

Despite the new insights that our study provides, there are some limitations that need to be acknowledged. First, due to its monocentric study design, external validity may be limited, and replication of our findings in a multicentric setting is warranted. Second, our analyses were performed in a mixed group of gastrointestinal cancer patients. Because of the small number of certain cancer types, this study cannot answer whether our results apply to all gastrointestinal tumor entities equally. Moreover, the study design limits our findings to the observation of associations. To clarify whether nutritional status has a causal effect on quality of life and fatigue, an interventional study using a standardized approach of providing nutritional support is required. Last, we cannot entirely rule out that minor further nutritional decline during the follow-up period resulted from selection bias. However, as we found no differences in baseline characteristics between study completers and dropouts, this seems unlikely.

## Conclusion

Most patients with gastrointestinal cancer are affected by malnutrition already at the time of diagnosis. Patients’ impaired nutritional status is closely linked with reduced quality of life and fatigue, especially their physical components. While minor further nutritional deterioration occurs during the early phase of treatment, these changes warrant attention, as especially the onset of cachexia and physical fatigue seem closely linked. Nutritional counseling and intervention must therefore be initiated as early as possible. Adequately powered, randomized controlled trials are desired to test whether optimized nutritional support beginning with diagnosis improves clinical outcome and experience along the patient’s journey.

## Supplementary Material

oyag028_Supplementary_Data

## Data Availability

The data underlying this article cannot be shared publicly due to ethical and legal considerations. The data will be shared on reasonable request to the corresponding author.
