# Supplementary material for: Malnutrition in gastrointestinal cancer manifests before systemic therapy and is associated with fatigue and reduced physical quality of life
Source: Oncologist. 2026 Feb 3;31(4):oyag028. doi: 10.1093/oncolo/oyag028 (PMC12988484; doi:10.1093/oncolo/oyag028)
Supplement: oyag028_Supplementary_Data [file oyag028_supplementary_data.zip › Supplementary Table 1.docx]

**Supplementary Table 1** Baseline routine and nutritional blood parameters.

|  |  | **Initial diagnosis**  **(n=29)** |  | **Undergoing chemotherapy**  **(n=37)** | **p-value** |
| --- | --- | --- | --- | --- | --- |
|  | Missing, n (%) |  | Missing, n (%) |  |  |
| **Complete blood Count** |  |  |  |  |  |
| Hemoglobin, mmol/L | 1 (3) | 7.20 (±1.32) | - | 7.03 (±1.12) | .582 |
| Hematocrit, L/L | 1 (3) | .36 (± .06) | - | .34 (±. 05) | .356 |
| Mean corpuscular volume, fL | 1 (3) | 89.18 (±5.50) | - | 88.48 (±6.83) | .658 |
| Mean corpuscular hematocrit, fmol | 1 (3) | 1.80 (± .14) | - | 1.81 (± .18) | .814 |
| Mean corpuscular hemoglobin concentration, mmol/L | 1 (3) | 20.15 (± .72) | - | 20.40 (±. 81) | .211 |
| Red blood cell count, 10^12^/L | 1 (3) | 4.01 (± .64) | - | 3.91 (±. 57) | .511 |
| White blood cell count, 10^9^/L | 1 (3) | 8.20 (5.42) | - | 4.70 (2.84) | **< .001** |
| Platelet count, 10^9^/L | 1 (3) | 278 (±112) | - | 181 (±92) | **< .001** |
| Mean platelet volume, fL | 1 (3) | 10.0 (2.3) | - | 10.1 (1.3) | .676 |
| **Blood chemistry** |  |  |  |  |  |
| Creatinine, µmol/L | 1 (3) | 81.4 (±24.8) | 1 (3) | 68.1 (±17.1) | **.014** |
| Blood urea nitrogen, mmol/L | 1 (3) | 5.6 (±2.3) | 2 (5) | 4.4 (±2.2) | **.047** |
| Total bilirubin, µmol/L | 1 (3) | 7.6 (6.2) | 1 (3) | 7.7 (5.0) | .636 |
| Direct bilirubin, µmol/L | 1 (3) | 2.8 (4.0) | 2 (5) | 2.4 (1.6) | .149 |
| Aspartate Aminotransferase, µkatal/L | 1 (3) | .70 (.92) | 1 (3) | .48 (.26) | .155 |
| Alanine aminotransferase, µkatal/L | 1 (3) | .60 (.89) | 1 (3) | .59 (.33) | .715 |
| Gamma-glutamyl transferase, µkatal/L | 1 (3) | 2.55 (8.27) | 1 (3) | 1.35 (3.28) | .201 |
| C-reactive protein, mg/L | 1 (3) | 37.1 (56.9) | 2 (5) | 6.1 (19.7) | **.001** |
| Albumin, g/L | 1 (3) | 29.0 (9.0) | 1 (3) | 32.0 (8.0) | .132 |

*Data are presented as mean (±SD) or median (IQR).*

*Differences between groups were tested by two-sided t-test or Mann–Whitney U test depending on the normality of data distribution.*
