# Supplementary material for: Malnutrition in gastrointestinal cancer manifests before systemic therapy and is associated with fatigue and reduced physical quality of life
Source: Oncologist. 2026 Feb 3;31(4):oyag028. doi: 10.1093/oncolo/oyag028 (PMC12988484; doi:10.1093/oncolo/oyag028)
Supplement: oyag028_Supplementary_Data [file oyag028_supplementary_data.zip › Supplementary Table 2.docx]

**Supplementary Table 2** Relation between nutritional status and fatigue subscales.

|  | **Total (n=65)** | | |  |
| --- | --- | --- | --- | --- |
|  | No  Malnutrition  (n=7) | Moderate Malnutrition  (n=27) | Severe Malnutrition  (n=31) | **p-value** |
| Physical fatigue | 22.9 (±11.5) | 47.2 (±30.0) | 61.3 (±23.5) | **0.002** |
| Emotional fatigue | 0.0 (22.2) | 22.2 (44.4) | 33.3 (44.4) | 0.137 |
| Cognitive fatigue | 0.0 (0.0) | 0.0 (33.3) | 0.0 (16.7) | 0.093 |
| Interference with daily life | 0.0 (33.3) | 0.0 (66.7) | 66.7 (100.0) | 0.054 |
| Social sequelae | 0.0 (0.0) | 0.0 (0.0) | 0.0 (0.0) | 0.229 |
| Total fatigue | 14.3 (±7.9) | 30.9 (±21.7) | 41.2 (±19.2) | **0.004** |
|  | **Initial Diagnosis (n=28)** | | |  |
|  | No  Malnutrition  (n=4) | Moderate Malnutrition  (n=11) | Severe Malnutrition  (n=13) | **p-value** |
| Physical fatigue | 23.3 (±8.6) | 53.3 (±33.7) | 60.4 (±27.0) | 0.094 |
| Emotional fatigue | 5.6 (44.4) | 33.3 (55.6) | 22.2 (44.4) | 0.550 |
| Cognitive fatigue | 0.0 (0.0) | 0.0 (33.3) | 0.0 (16.7) | 0.305 |
| Interference with daily life | 0.0 (25.0) | 66.7 (100.0) | 66.7 (83.3) | 0.147 |
| Social sequelae | 0.0 (0.0) | 0.0 (0.0) | 0.0 (50.0) | 0.418 |
| Total fatigue | 14.6 (±8.0) | 38.4 (±25.6) | 40.8 (±20.0) | 0.114 |
|  | **Undergoing chemotherapy (n=37)** | | |  |
|  | No  Malnutrition  (n=3) | Moderate Malnutrition  (n=16) | Severe Malnutrition  (n=18) | **p-value** |
| Physical fatigue | 22.2 (±16.8) | 42.9 (±27.5) | 61.9 (±21.4) | **0.014** |
| Emotional fatigue | 0.0 (22.2) | 11.1 (22.2) | 33.3 (27.8) | **0.046** |
| Cognitive fatigue | 0.0 (0.0) | 0.0 (16.7) | 8.3 (16.7) | 0.314 |
| Interference with daily life | 33.3 () | 0.0 (58.3) | 66.7 (100.0) | 0.061 |
| Social sequelae | 0.0 (0.0) | 0.0 (0.0) | 0.0 (0.0) | 0.338 |
| Total fatigue | 13.9 (±9.6) | 25.7 (±17.7) | 41.5 (±19.2) | **0.013** |

*All data is presented as mean (±SD) or median (IQR) depending on its distribution*

*Scores for total fatigue and each fatigue subscales range from 0 to 100*

*Differences between groups were tested using one-way analysis of variance or Kruskal-Wallis test for normally and non-normally distributed data, respectively.*
