# Supplementary material for: Malnutrition in gastrointestinal cancer manifests before systemic therapy and is associated with fatigue and reduced physical quality of life
Source: Oncologist. 2026 Feb 3;31(4):oyag028. doi: 10.1093/oncolo/oyag028 (PMC12988484; doi:10.1093/oncolo/oyag028)
Supplement: oyag028_Supplementary_Data [file oyag028_supplementary_data.zip › Supplementary Table 3.docx]

**Supplementary Table 3** Comparison of routine and nutritional blood parameters at baseline and after 3 months.

|  |  | **Baseline (n=36)** | **After 3 months (n=36)** | **p-value** |
| --- | --- | --- | --- | --- |
|  | Missing, n (%) |  |  |  |
| **Complete blood Count** |  |  |  |  |
| Hemoglobin, mmol/L |  | 7.13 (±1.11) | 7.19 (±1.03) | .668 |
| Hematocrit, L/L |  | .35 (± .05) | .35 (± .04) | .659 |
| Mean corpuscular volume, fL |  | 88.74 (±6.36) | 89.31 (±7.46) | .479 |
| Mean corpuscular hematocrit, fmol |  | 1.80 (± .17) | 1.82 (± .20) | .394 |
| Mean corpuscular hemoglobin concentration, mmol/L |  | 20.35 ( .90) | 20.20 (1.30) | .915 |
| Red blood cell count, 10^12^/L |  | 3.97 (± .53) | 3.98 (± .52) | .891 |
| White blood cell count, 10^9^/L |  | 5.71 (4.32) | 5.48 (3.11) | .671 |
| Platelet count, 10^9^/L |  | 210 (±98) | 194 (±88) | .301 |
| Mean platelet volume, fL |  | 10.4 (± .9) | 10.2 (± .9) | .297 |
| **Blood chemistry** |  |  |  |  |
| Creatinine, µmol/L | 1 (3) | 71.1 (±18.3) | 72.1 (±15.7) | .736 |
| Blood urea nitrogen, mmol/L | 2 (6) | 4.9 (±2.2) | 4.6 (±1.2) | .359 |
| Total bilirubin, µmol/L | 1 (3) | 6.9 (4.6) | 6.0 (6.7) | .620 |
| Direct bilirubin, µmol/L | 2 (6) | 2.35 (1.30) | 2.20 (1.60) | .673 |
| Aspartate Aminotransferase, µkatal/L | 1 (3) | .50 (.36) | .52 (.45) | .210 |
| Alanine aminotransferase, µkatal/L | 1 (3) | .59 (.57) | .62 (.54) | .932 |
| Gamma-glutamyl transferase, µkatal/L | 1 (3) | 1.30 (2.71) | 1.10 (2.03) | .235 |
| C-reactive protein, mg/L | 1 (3) | 6.2 (19.7) | 5.1 (37.7) | .446 |
| Albumin, g/L | 2 (6) | 31.4 (±3.8) | 31.7 (±4.8) | .722 |

*Data are presented as mean (±SD) or median (IQR).*

*Differences between time points were tested by paired samples Wilcoxon or t-test depending on the normality of data distribution.*
