# Supplementary material for: Malnutrition in gastrointestinal cancer manifests before systemic therapy and is associated with fatigue and reduced physical quality of life
Source: Oncologist. 2026 Feb 3;31(4):oyag028. doi: 10.1093/oncolo/oyag028 (PMC12988484; doi:10.1093/oncolo/oyag028)
Supplement: oyag028_Supplementary_Data [file oyag028_supplementary_data.zip › Supplementary Table 4.docx]

**Supplementary Table 4** Comparison of baseline demographic and clinical patient characteristics between completers and dropouts

|  | | **Completers**  **(n=36)** | **Dropouts**  **(n=30)** | **p-value** |
| --- | --- | --- | --- | --- |
| Age, years | | 60.9 (±10.3) | 63.6 (±10.8) | 0.319 |
| Male, n (%) | | 23 (64) | 22 (73) | 0.412 |
| Study group, n (%) | |  |  | **0.004** |
|  | Initial diagnosis | 10 (28) | 19 (63) |  |
|  | Undergoing systemic therapy | 26 (72) | 11 (37) |  |
| Tumor entity, n (%) | |  |  |  |
|  | Pancreas | 13 (36) | 8 (27) | 0.449 |
|  | Colorectal | 13 (36) | 10 (33) |  |
|  | Gastric | 6 (17) | 4 (13) |  |
|  | Esophagus | 2 (6) | 2 (7) |  |
|  | Liver | - | 2 (7) |  |
|  | Bile duct | 1 (3) | 4 |  |
|  | Neuroendocrine | 1 (3) | - |  |
| Metastasized, n (%) | | 18 (50) | 20 (67) | 0.173 |
| Treatment intention, n (%) | |  |  | **0.045** |
|  | Curative | 17 (47) | 7 (23) |  |
|  | Palliative | 19 (53) | 23 (77) |  |
| ECOG performance status, n (%) | |  |  | 0.451 |
|  | 0 | 17 (47) | 11 (37) |  |
|  | I | 14 (39) | 10 (33) |  |
|  | II | 4 (11) | 6 (20) |  |
|  | III | 1 (3) | 3 (10) |  |
| Time since initial diagnosis, months | | 2.8 (5.9) | 1.1 (2.7) | **0.035** |

*Data are presented as mean (±SD), median (IQR), or n (%)*

*Differences between groups for continuous data were tested by two-sided t-test or Mann–Whitney U test depending on the normality of data distribution.*

*Differences between groups for categorical data were tested by Chi-squared or Fisher’s exact test.*
