# Supplementary material for: Malnutrition in gastrointestinal cancer manifests before systemic therapy and is associated with fatigue and reduced physical quality of life
Source: Oncologist. 2026 Feb 3;31(4):oyag028. doi: 10.1093/oncolo/oyag028 (PMC12988484; doi:10.1093/oncolo/oyag028)
Supplement: oyag028_Supplementary_Data [file oyag028_supplementary_data.zip › Supplementary Table 5.docx]

**Supplementary Table 5** Comparison of baseline nutritional status between completers and dropouts

|  | | **Completers**  **(n=36)** | **Dropouts**  **(n=30)** | **p-value** |
| --- | --- | --- | --- | --- |
| Nutritional Risk | |  |  |  |
|  | NRS-2002, pts | 3 (2) | 3 (2) | 0.785 |
|  | NRS-2002 ≥ 3 pts, n (%) | 20 (56) | 18 (60) | 0.716 |
| Reduced food intake, n (%) | | 18 (50) | 24 (80) | **0.012** |
| Malassimilation/ Chronic gastrointestinal condition, n (%) | | 11 (31) | 10 (33) | 0.809 |
| Low body mass index, n (%) | | 6 (17) | 5 (17) | 1.000 |
| Weight loss, n (%) | | 26 (72) | 24 (80) | 0.629 |
| Reduced muscle mass, n (%) | | 19 (53) | 13 (43) | 0.445 |
| Malnutrition, n (%) | |  |  | 0.253 |
|  | No | 4 (11) | 4 (13) |  |
|  | Moderate | 18 (50) | 9 (30) |  |
|  | Severe | 14 (39) | 17 (57) |  |
| Cachexia, n (%) | | 17 (42) | 24 (80) | **0.006** |
| Sarcopenia^1^, n (%) | | 0 (0) | 1 (4) | 0.437 |

*Data are presented as n (%) or median (IQR)*

*Differences between groups for continuous data were tested by Mann–Whitney U test.*

*Differences between groups for categorical data were tested by Chi-squared or Fisher’s exact test.*

^1^ n=28 for dropouts due to missing values
