# Supplementary material for: Malnutrition in gastrointestinal cancer manifests before systemic therapy and is associated with fatigue and reduced physical quality of life
Source: Oncologist. 2026 Feb 3;31(4):oyag028. doi: 10.1093/oncolo/oyag028 (PMC12988484; doi:10.1093/oncolo/oyag028)
Supplement: oyag028_Supplementary_Data [file oyag028_supplementary_data.zip › Supplementary Table 6.docx]

**Supplementary Table 6** Comparison of baseline body weight, composition, and weight loss between completers and dropouts

|  | **Completers**  **(n=36)** | **Dropouts**  **(n=30)** | **p-value** |
| --- | --- | --- | --- |
| Body mass index, kg/m^2^ | 25.4 (±4.7) | 24.8 (±4.5) | 0.554 |
| Fat mass index, kg/m^2^ | 6.0 (5.3) | 4.8 (3.0) | 0.345 |
| Skeletal muscle mass index, kg/m^2^ | 8.4 (3.1) | 8.3 (2.3) | 0.923 |
| Phase angle, ° | 5.1 (±0.9) | 4.5 (±1.2) | 0.055 |
| Past 6 months weight loss, % | 5.1 (11.3) | 8.7 (9.5) | **0.047** |
| Total weight loss, % | 11.7 (12.6) | 12.2 (8.4) | 0.877 |

*Data are presented as mean (±SD) or median (IQR).*

*Differences between groups were tested by two-sided t-test or Mann–Whitney U test depending on the normality of data distribution.*

*Body composition parameters: n=27 for dropouts*
