# Supplementary figures and images for: Malnutrition in gastrointestinal cancer manifests before systemic therapy and is associated with fatigue and reduced physical quality of life
Source: Oncologist. 2026 Feb 3;31(4):oyag028. doi: 10.1093/oncolo/oyag028 (PMC12988484; doi:10.1093/oncolo/oyag028)

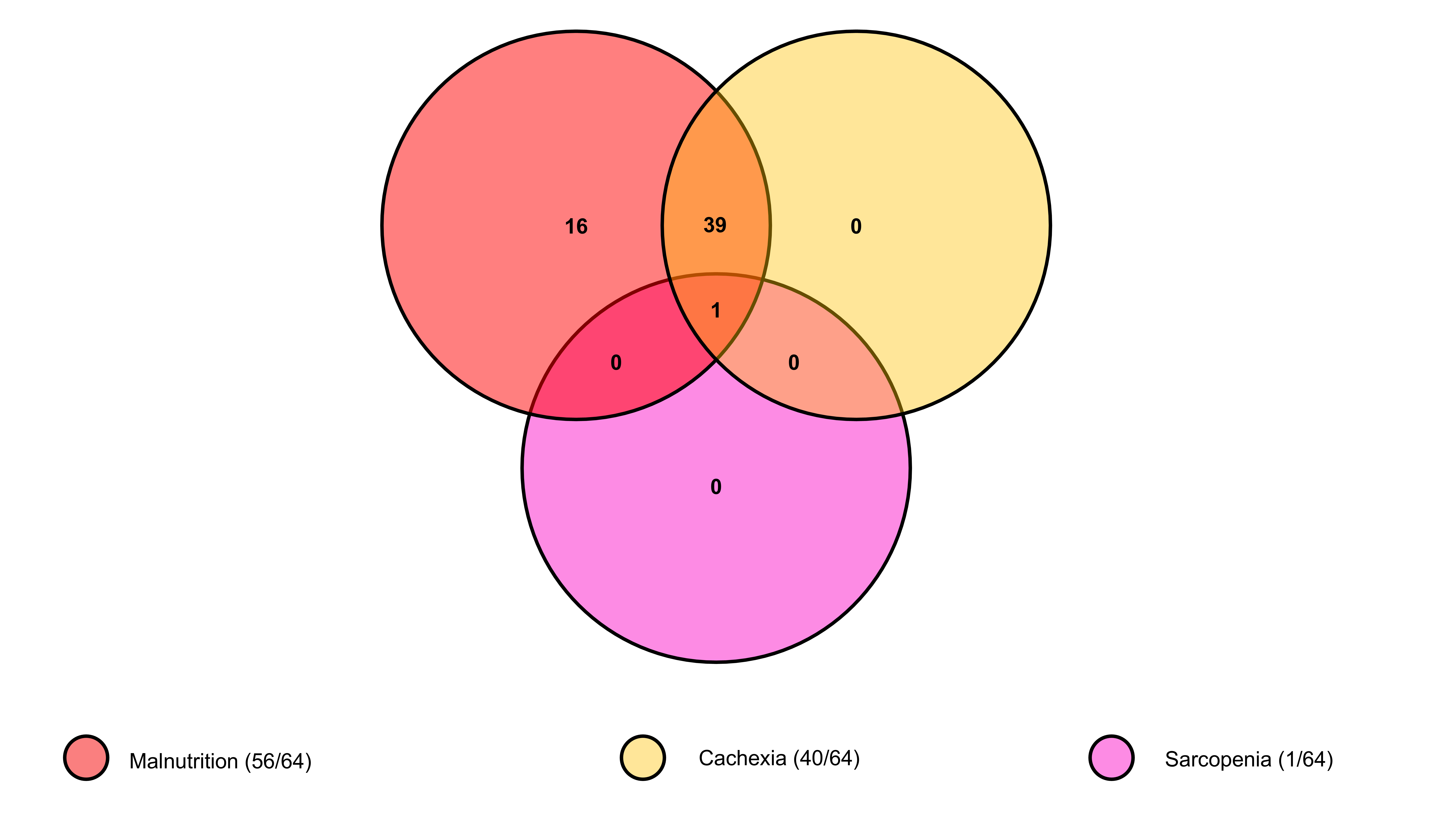

Supplement: oyag028_Supplementary_Data [file oyag028_supplementary_data.zip › Supplemenatry Figure 2 600DPI.TIF]
